# Prevalence and associated factors of HIV self-testing among men who have sex with men in Ningbo, China: a cross-sectional study

**DOI:** 10.1186/s12981-021-00339-x

**Published:** 2021-04-20

**Authors:** Hang Hong, Hong-bo Shi, Hai-bo Jiang, Hong-jun Dong, Yun-liang Shen

**Affiliations:** 1 Ningbo Municipal Center for Disease Control and Prevention, 237 Yongfeng Road, Ningbo, 315010 Zhejiang China; 2 Zhejiang Provincial Institute of Dermatology, 61 Wuyuan Road, Deqing, 313200 Zhejiang China

**Keywords:** MSM, HIV self-testing, Gay apps, Associated factors, China

## Abstract

**Background:**

HIV testing and early linkage to care are critical for reducing the risk of HIV transmission. HIV self-testing (HIVST) is a useful tool for increasing HIV testing frequency.This study aimed to investigate HIVST rates among men who have sex with men (MSM), the characteristics of MSM who had HIVST, and factors associated with HIVST uptake among MSM in Ningbo, China.

**Methods:**

A cross-sectional study was conducted from April to October 2019 in Ningbo,China. Participants were aged at least 18 years and having had sexual contact with men in the past year. Proportions were used for categorical variables. Adjusted Odds Ratio (AOR) and 95% Confidence Interval (CI) for characteristics associated with HIVST uptake was processed by multivariable logistic regression models.

**Results:**

Among a sample of 699 MSM recruited, 38.2% had reported previous use of an HIV self-test kit. A greater proportion of HIVST users had a higher frequency of HIV testing (≥ 2 times: 70.0% versus 41.2%, p < 0.001) in the past 1 year. The odds of older age (30–39 years: AOR = 0.49, CI 0.32–0.76; more than 40 years: AOR = 0.07, CI 0.04–0.14, compared to 18–29 years), bisexual (AOR = 0.49, CI 0.29–0.84) were lower among HIVST users,and were higher among MSM who were higher education level (high school: AOR = 2.82, CI 1.70–4.69, compared to middle school or less), gay apps use (AOR = 1.86, CI 1.13–3.05), multiple male sex partners (AOR = 1.90, CI 1.29–2.80), frequency of male–male sexual contact ≥ 1 times per week (AOR = 1.86, CI 1.30–2.66), syphilis infection (AOR = 5.48, CI 2.53–11.88).

**Conclusions:**

Further HIVST education should be strengthened for school-aged children and teenagers, and free HIVST kits may be provided to high-risk MSM through gay apps and CBO to achieve the increased HIV testing frequency.

## Introduction

Globally, there were an estimated 37.9 million people are living with HIV (PLWH), with about 1.7 million people newly infected with HIV at the end of 2018 [1]. Men who have sex with men (MSM) has become the high-risk group of HIV acquisition [2, 3]. MSM accounted for an estimated 17% of new HIV infections globally, including more than half of new HIV infections in western and central Europe and North America [4]. MSM was about 28 times more likely to be living with HIV than it was among all adult men in 2018 [5].

To end the AIDS epidemic by 2030, the "90-90-90" goal by 2020 set up by UNAIDS in 2014 (90% of people with HIV infection diagnosed, 90% of people diagnosed on treatment, and 90% of people on treatment achieving virological suppression) [6]. However, it seems unlikely that many regions and countries would reach the target, especially the first 90% [7]. In China, an estimated less than 70% of PLWH were aware of their HIV-positive status by the end of 2018 [8]. HIV testing and early linkage to care were critical for reducing the risk of viral transmission from infected persons However, key population groups including MSM were unwilling to seek voluntary HIV counseling and testing (VCT) in the hospital or Centers for Disease Control and Prevention (CDC) due to stigma and discrimination [9–11].

HIV self-testing (HIVST) had recommended being offered as an additional HIV testing approach by WHO in 2016 [12]. Testers take their blood sample to perform HIV rapid tests and interpret the result at the time and location of their choosing. Several studies showed that HIVST had generally high sensitivity and specificity and was an acceptable and feasible testing approach due to the convenience, privacy, and ease of use [13–15]. These characteristics make it a potentially useful tool for increasing testing frequency and easy to reach first time and repeat testers for HIV [16, 17]. In China, HIVST is highly acceptable and easily available through drugstore, e-commerce platform and community-based organisations (CBO) [18].

Given the need to improve HIV testing rates and target the first of the United Nation’s 90-90-90 HIV testing and treatment goals, the purpose of this study was to investigate HIVST uptake rates among MSM, the characteristics and factors associated with HIVST uptake among MSM in Ningbo, China.

## Methods

### Study design and participants

We conducted a cross-sectional survey from April 1 to October 30, 2019, in Ningbo. Ningbo is an eastern coastal city of China, nearby Shanghai, with an area of 9365 km^2^ and a population of approximately 8.54 million people. HIV prevalence among MSM in Ningbo was 5.7% [19].Convenience sampling of participants was recruited through a combined online and offline method. Flyer advertisements were posted in MSM venues (Three parks, two bars, and eight community events) and VCT clinic,as well as on gay websites and gay apps. The criteria for recruiting were (1) being male, (2) aged at least18 years, (3) having resided in Ningbo for at least 6 months, (4) having had sexual contact with men in the past year.

Interested MSM contacted trained project workers for assessment of eligibility. After providing written informed consent, eligible participants were asked to complete self-administered questionnaires. Project workers were instructed to check questionnaires in place to ensure collection of quality data.

### Questionnaire

All data were collected through self-administered paper questionnaires by trained project workers. The following variables were included: (1) demographic information including age, marital status, education level, duration of local residence, monthly income, and sexual orientation; (2) gay apps (Blued, Jack’d, and ZANK) use including duration and frequency of apps use; (3) Sexual behaviors including role and frequency in sexual intercourse, multiple male sex partners, unprotected sex with men and syphilis infection in the past 6 months; (4) HIV testing including reasons for test, frequency of test, time since latest test and site of the latest test; (5) HIVST including self-reported used HIVST in the lifetime (HIVST users), the type of HIVST kit,way to receive HIVST kit.

### Statistical analysis

Characteristics of all participants were described by categorical variables presented as absolute values and percentages. The demographic information, gay apps use, sexual behaviors and HIV testing compared between HIVST users and Non-HIVST users were examined by chi-square tests. Univariate and multivariable forward stepwise logistic regression models were performed to examine risk factors associated with HIVST. The statistical significance was defined as P < 0.05. All statistical analyses were performed in SPSS (version 21.0, IBM, Armonk, NY, USA).

### Ethical considerations

This study protocol was reviewed and approved by the Institutional Review Board of the Ningbo CDC. Informed consent was asked to sign for all eligible participants when the survey was starting. Participants could receive a gift for prizes of up to 50 Chinese Yuan (CNY) upon the completion of the survey.

## Results

### Participant characteristics

Table 1 demonstrates the characteristics of the 699 MSM in Ningbo. The mean age was 31.9 (SD 8.8) years. Most participants (81.1%) were less than 30 years, 63.4% were single, 70.4% had a high school education or above, 77.7% had lived in Ningbo for at least 2 years, 62.9% had an income above 5000 CNY per month, and 74.8% self-identified as gay.

**Table 1:** Characteristics of study participants and of HIVST users and Non-HIVST users in Ningbo, China, 2019

Characteristics	All participants n (%)	Subgroup comparison		
		HIVST users n (%)	Non-HIVST users n (%)	P-value^c^
Overall	699 (100)	267 (100)	432 (100)	
Demographics				
Age (years)				
18 ~	330 (47.2)	155 (58.1)	175 (40.5)	< 0.001
30 ~	237 (33.9)	92 (34.5)	145 (33.6)	
≥ 40	132 (18.9)	20 (7.5)	112 (25.9)	
Marital status				
Single	443 (63.4)	172 (64.4)	271 (62.7)	0.653
Married	256 (36.6)	95(35.6)	161 (37.3)	
Education level				
Middle school or less	207 (29.6)	65 (24.3)	142 (32.9)	0.022
High school	264 (37.8)	101 (37.8)	163 (37.7)	
College or above	228 (32.6)	101 (37.8)	127 (29.4)	
Duration of local residence (years)				
< 2	156 (22.3)	76 (28.5)	80 (18.5)	0.002
≥ 2	543 (77.7)	191 (71.5)	352 (81.5)	
Monthly income (CNY)				
< 5000	259 (37.1)	74 (27.7)	185 (42.8)	< 0.001
≥ 5000	440 (62.9)	193 (72.3)	247 (57.2)	
Sexual orientation				
Gay	523 (74.8)	220 (82.4)	303 (70.1)	0.001
Bisexual	135 (19.3)	34 (12.7)	101 (23.4)	
Unknown/unsure	41 (5.9)	13 (4.9)	28 (6.5)	
Gay apps usage				
Gay apps use				
Yes	583 (83.4)	237 (88.8)	346 (80.1)	0.003
No	116 (16.6)	30 (11.2)	86 (19.9)	
Duration of gay apps use (years)				
< 1	83 (14.2)	25 (10.5)	58 (16.8)	0.035
≥ 1	500 (85.8)	212 (89.5)	288 (83.2)	
Missing (No use of gay apps)	116			
Frequency of gay apps use (times/day)^a^				
< 1	66 (11.3)	22 (9.3)	44 (12.7)	0.036
1-	185 (31.7)	65 (27.4)	120 (34.7)	
≥ 5	332 (56.9)	150 (63.3)	182 (52.6)	
Missing (No use of gay apps)	116			
Sexual behavior				
Role in sexual intercourse				
Insertive anal sex	191 (27.3)	74 (27.7)	117 (27.1)	0.983
Receptive anal sex	155 (22.2)	59 (22.1)	96 (22.2)	
Both	353 (50.5)	134 (50.2)	219 (50.5)	
Frequency of male–male sexual contact (times/week)^a^				
< 1	377 (53.9)	124 (46.4)	253 (58.6)	0.002
≥ 1	322 (46.1)	143 (53.6)	179 (41.4)	
Multiple male sex partners^a^				
Yes	196 (28.0)	87 (32.6)	109 (25.2)	0.035
No	503 (72.0)	180 (67.4)	323 (74.8)	
Unprotected sex with men^a^				
Yes	190 (27.2)	64 (24.0)	126 (29.2)	0.133
No	509 (72.8)	203 (76.0)	306 (70.8)	
Syphilis Infection^b^				
Yes	44 (6.3)	29 (10.9)	15 (3.5)	< 0.001
No	655 (93.7)	238 (89.1)	417 (96.5)	
HIV testing				
Reason for HIV testing				
Regular HIV testing	432 (71.5)	196 (73.4)	236 (70.0)	0.011
Had unprotected sexual behavior	60 (9.9)	22 (8.2)	38 (11.3)	
Had suspicions symptoms of AIDS	47 (7.8)	19 (7.1)	28 (8.3)	
Awareness of HIV testing results before sex	36 (6.0)	10 (3.7)	26 (7.7)	
Repeated HIV testing	29 (4.8)	20 (7.5)	9 (2.7)	
Missing(Had never HIV testing before)	95			
Frequency of HIV testing^b^				
0	114 (16.3)	4 (1.5)	110 (25.5)	< 0.001
1	220 (31.5)	76 (28.5)	144 (33.3)	
≥ 2	365 (52.2)	187 (70.0)	178 (41.2)	
Time since latest HIV testing (months)				
1 ~	148 (24.5)	81 (30.3)	67 (19.9)	0.005
4 ~	176 (29.1)	77 (28.8)	99 (29.4)	
7 ~	92 (15.2)	29 (10.9)	63 (18.7)	
13 ~	188 (31.1)	80 (30.0)	108 (32.0)	
Missing(Had never HIV testing before)	95			
Site of the latest HIV testing				
CDC	211 (34.9)	85 (31.8)	126 (37.4)	0.029
Hospital	43 (7.1)	13 (4.9)	30 (8.9)	
NGO	304 (50.3)	169 (63.3)	181 (53.4)	
Missing (Had never HIV testing before)	95			

^a^In the prior 6 months

^b^In the prior one year

^c^Subgroups were compared using chi-square tests to generate P-

Most participants (83.4%) had used gay apps in the past 6 months. Of the 583 gay app users, 85.8% (500/583) had used gay apps for at least 1 year, 56.9% (332/583) used them at least 5 times a day. In terms of sexual practice, half of the participants were engaged in both insertive anal intercourse and receptive anal intercourse equally. 46.1% had sex with men for at least once per week in the prior 6 months.72.0% had multiple male sex partners and 27.2% had unprotected sex with men in the prior 6 months.

### Comparisons of characteristics between HIVST users and Non-HIVST users

Among all participants, 604 (86.4%) reported having HIV testing at least once in their lifetimes, and 575 (82.3%) had been tested in the past year. Table 1 describes that a total of 267 participants of (699, 38.2%) reported having used an HIV self-test kit before, whereas 432 (61.8%) reported never having HIV self-test. Compared to non-HIVST users, a larger proportion of HIVST users were aged between 18 and 29 years (58.1% versus 40.5%, p < 0.001), had college or above education level (37.8% versus 29.4%, p = 0.022), had lived in Ningbo less than 2 years (28.5% versus 18.5%, p = 0.002), had an income above 5000 China Yuan (CNY) per month (72.3% versus 57.2%, p < 0.001) and self-identified as gay (82.4% versus 70.1%, p < 0.001).

A larger proportion of HIVST users reported having higher frequency of male-male sexual contact (≥ 1 time/week: 53.6% versus 41.4%, p = 0.002), having multiple male sex partners (32.6% versus 25.2%, p = 0.035), having had syphilis infection (10.9% versus 3.5%, p < 0.001) and having used gay apps (88.8% versus 80.1%, p = 0.003) in the prior 6 months. Among those who had used gay apps, a greater proportion of HIVST users also had a higher frequency of gay apps use (≥ 5 times/day: 63.3% versus 52.6%, p = 0.036) in the prior 6 months and used gay apps over 1 year (89.5% versus 83.2%, p = 0.035).

A greater proportion of HIVST users had a higher frequency of HIV testing (≥ 2 times: 70.0% versus 41.2%, p < 0.001) in the past 1 year. Among those who had HIV testing before, a greater proportion of HIVST users reported having HIV testing regularly (73.4% versus 70.0%, p = 0.011), their most recent HIV testing had been within the prior 3 months (30.3% versus 19.9%, p = 0.005), the site of latest HIV testing was CBO (63.3% versus 53.4%, p = 0.029).

### Factor associated with HIVST uptake

As show in Table 2, multivariable logistic regression analyses found that the odds of older age (30–39 years: AOR = 0.49, CI 0.32–0.76; more than 40 years: AOR = 0.07, CI 0.04–0.14, compared to 18–29 years), bisexual (AOR = 0.49, CI 0.29–0.84) were lower among HIVST users,and were higher among MSM who were higher education level (high school: AOR = 2.82, CI 1.70–4.69, compared to middle school or less), gay apps use (AOR = 1.86, CI 1.13–3.05), multiple male sex partners (AOR = 1.90, CI 1.29–2.80), frequency of male–male sexual contact ≥ 1 times per week (AOR = 1.86, CI 1.30–2.66), syphilis infection (AOR = 5.48, CI 2.53–11.88).

**Table 2:** Factors associated with HIVST among study participants in Ningbo, China, 2019 (n = 699)

Factor	Unadjusted OR (CI)^b^	P-value	Adjusted OR (CI)^b^	P-value
Age (years)				
18 ~	1.00		1.00	
30 ~	0.72 (0.51–1.01)	0.054	0.49 (0.32–0.76)	0.001
≥ 40	0.20 (0.12–0.34)	< 0.001	0.07 (0.04–0.14)	< 0.001
Marital status				
Married	1.00		–	
Single	1.08 (0.78–1.48)	0.653	–	–
Education level				
Middle school or less	1.00		1.00	
High school	1.74 (1.17–2.57)	0.006	2.82 (1.70–4.69)	< 0.001
College or above	1.35 (0.92–1.99)	0.123	1.42 (0.92–2.20)	0.052
Local residence time				
< 2	1.00		1.00	
2	0.57 (0.40–0.82)	0.002	0.66 (0.44–1.00)	0.052
Monthly income (CNY)				
< 5000	1.00		1.00	
≥ 5000	1.95 (1.41–2.71)	< 0.001	1.35 (0.94–1.93)	0.100
Sexual orientation				
Gay	1.00		1.00	
Bisexual	0.46 (0.30–0.71)	< 0.001	0.49 (0.29–0.84)	0.009
Unknown/unsure	0.64(0.32–1.26)	0.198	0.71 (0.34–1.51)	0.379
Gay apps use				
No	1.00		1.00	
Yes	1.96 (1.26–3.07)	0.003	1.86 (1.13–3.05)	0.014
Multiple male sex partners^a^				
No	1.00		1.00	
Yes	1.43 (1.02–2.00)	0.036	1.90 (1.29–2.80)	0.001
Frequency of male–male sexual contact (times/week)^a^				
< 1	1.00		1.00	
≥ 1	1.63 (1.20–2.22)	0.002	1.86 (1.30–2.66)	0.001
Unprotected sex with men^a^				
No	1.00		–	
Yes	0.77 (0.54–1.09)	0.134	–	–
Syphilis infection				
No	1.00		1.00	
Yes	3.39 (1.78–6.45)	< 0.001	5.48 (2.53–11.88)	< 0.001

^a^In the prior 6 months

^b^Univariate and multivariate regression analyses were used to generate odds ratios (ORs) and 95% confidence intervals (CIs)

## Discussion

Globally, HIV testing had become an important strategy to end the HIV epidemic [20, 21]. HIVST is reliable, safe, and accurate, which can help increase serostatus awareness and ultimately linkage-to-care or prevention services among HIV high-risk populations [22, 23]. The proportion of HIV testing among MSM in the lifetimes and the past year in our analysis was higher than in other studies, but there is a certain distance to reach the first 90% targets by 2020 [7, 21]. The study revealed that 38.2% of MSM had used HIVST before in Ningbo, which was lower than the rates reported in studies from other areas [24, 25]. The reasons for the relatively low HIVST rate in our study could be related to the lack of inventions to promote HIVST by Ningbo CDC. Our study’s contribution to investigate factors associated with HIVST and help the government develop targeted strategies to improve HIV testing among MSM in China.

Our study showed that those MSM who were younger or high education levels were more likely to have had HIVST. It is possible that younger MSM had more worries about positive test results [11]. Worldwide, about 32% of new HIV infections among adults aged 15 years and older have occurred in youth ages 15–24 years in 2018 [4]. Therefore, HIVST education should be included as a part of comprehensive sexual and reproductive health education for school-age children and teenagers [23].

Gay apps were very popular among MSM in China [19]. HIV prevention through gay apps was widely applied toward reducing high-risk behaviors and promoting HIV testing [26, 27]. Our results showed that HIVST users had a higher frequency of gay apps use than Non-HIVST users in the prior 6 months. As with previous findings, the utility of mobile health interventions can engage MSM in HIVST in Heifei and Shenzhen, China [28, 29], and increase rates of confirmed HIV diagnoses and linkage to clinical care in the UK [30]. It indicated that HIVST kits usage and offer can be conducted as a part of HIV prevention through gay apps to access to more high-risk populations in China.

Furthermore, consistent with findings in other studies [24], those who had high-risk sex behavior, including multiple male sex partners, frequency of male-male sexual contact more than once per week and syphilis infection were more likely to have had HIVST. It is possible that commercial HIVST kits can be easily bought by online shopping platform in China. These high-risk MSM would be willingness to pay for HIVST kits instead of testing in the hospital [24]. But to avoid possible cost barriers, free HIVST kits might be provided to high-risk populations to achieve the increased testing frequency^17^.

This study demonstrated that HIVST users were more like to have a higher frequency of HIV testing and regular HIV testing compared to non-HIVST users. Regular HIV testing enables early identification and treatment of HIV among at-risk MSM [31]. As mentioned, the US CDC recommends MSM to take up HIV testing every 3–6 months if they have additional HIV risk factors [32]. But most of these MSM have no HIV testing routines [33]. So some HIV interventions should be improved to encourage MSM to use HIVST regular after VCT or HIV risk assessment. The results also showed that two-thirds of MSM received HIVST kits form CBO in the latest HIV testing. This indicated that CBO had become an important role in HIV intervention in China. So the government might strengthen support to CBO to promote HIVST uptake.

## Limitations

The present study had several limitations. First, participants were relatively high-educated, had higher income, and our findings may not be generalizable in other contexts or settings. Second, some questions were asked in the prior 6 months. Despite implemented quality control measures, recall and social desirability bias might have existed.Third, this was not a representative sample of the MSM population in Ningbo, because only those who had a willingness and contacted trained project workers could be recruited into the study.Finally, as this was a cross-sectional study, we are unable to establish a causal relationship.

## Conclusions

The coverage of HIVST had a significant gap in Ningbo, China. It is necessary to make continued efforts to expand HIVST coverage among MSM. Further HIVST education should strengthen for school-age children and teenagers, HIV prevention should include HIVST kits usage and offer through gay apps and CBO, and free HIVST kits might be provided to high-risk MSM to accelerate achieve the "90-90-90" goal.

## Data Availability

The datasets used and/or analyzed during this study is not publicly available, but may be available from the corresponding author upon reasonable request, and with permission from Ningbo Municipal Center for Disease Control and Prevention.

## Abbreviations

HIVST: HIV self-testing
PLWH: People are living with HIV
VCT: Voluntary HIV counseling and testing
CDC: Centers for disease control and prevention
